# Bilateral Asymmetries in Ultrasound Assessments of the Rectus Femoris throughout an NCAA Division I Volleyball Preseason

**DOI:** 10.3390/sports6030094

**Published:** 2018-09-12

**Authors:** Gabriel J. Sanders, Brian Boos, Frank Shipley, Corey A. Peacock

**Affiliations:** 1 Department Kinesiology and Health, Northern Kentucky University, Highland Heights, KY 41099, USA; 2 Department of Strength and Conditioning, Northern Kentucky University, Highland Heights, KY 41099, USA; boosb1@nku.edu; 3 Department of Sports Medicine, Northern Kentucky University, Highland Heights, KY 41099, USA; shipleyf1@nku.edu; 4 Department Exercise and Sport Science, Nova Southeastern University, Fort Lauderdale, FL 33314, USA; cpeacock@nova.edu

**Keywords:** glycogen, ultrasound, athlete monitoring, muscle fuel rating, performance

## Abstract

The purpose of the study was to assess glycogen content of the rectus femoris (RF) muscles utilizing high-frequency ultrasound throughout an intensive, nine-day preseason training period in NCAA division I volleyball athletes. In the morning prior to the beginning of practice, athletes (n = 13) left and right RF muscles were assessed via ultrasound to quantify muscle fuel ratings (0–100 score range). The recommended location of the RF ultrasound scans were based on manufacturer guidelines, and the same technician recorded the daily measurements. To assess daily training load, session ratings of perceived exertion (s-RPE) were utilized. A paired *t*-test revealed a large significant difference between left (51.7 ± 17.9) and right (32.8 ± 17.4) RF muscle fuel ratings (*p* < 0.001). There was also a major effect of time on s-RPE (*p* < 0.001) and left (dominant) RF fuel rating (*p* = 0.001). s-RPE decreased from the beginning to the end of the training camp. However, left RF fuel ratings increased from the first to the second day, then remained elevated all throughout the preseason. In conclusion, all athletes were left-leg dominant and had a 57.6% bilateral asymmetry between their left and right RF muscle fuel ratings despite changes in training load. High-frequency ultrasounds are a noninvasive assessment tool that can determine glycogen replenishment asymmetries in the RF.

## 1. Introduction

Traditionally, muscle biopsies are needed to assess muscle glycogen levels [1]. Biopsies are invasive, painful, and not feasible to use on athletes when they are preparing for a season [2]. However, a noninvasive approach to quantify glycogen content in the muscles exists via high-frequency ultrasound technology. Ultrasounds have been shown to detect both hydration status and muscle glycogen content [3,4]. Additionally, ultrasound technology has been found to correlate well with histochemical analyses of rectus femoris (RF) muscle biopsies [3]. Therefore, ultrasound is a potentially useful tool to incorporate in athlete monitoring protocols to measure glycogen content of the muscle [2] as an indicator of lower substrate availability, recovery, and dietary habits.

Volleyball is an intermittent sport with significant anaerobic components that require repetitive jumps to successfully participate in practice and competitions [5,6,7,8]. Volleyball athletes can accumulate more than 44 high-intensity jumps in games and more than 115 high-intensity jumps in high-volume practices [9]. In addition to jump loads, session (s-RPE) ratings of perceived exertion have been used to quantify internal training load and can exceed 1334 arbitrary units (AU) during a five-set match [10]. A five-set match is physically demanding; however, it is reasonable to suggest that preseason training, at times, imposes a greater training load than a five-set match. Preseason training camps require athletes to participate in multiple daily practice sessions, in addition to conditioning and strength sessions, and it is unknown how these training loads affect fatigue and muscle glycogen replenishment.

Excessive training loads in a volleyball preseason training camp may significantly alter muscle contractile properties, stretch-shortening capabilities, and even glycogen stores, primarily if athletes are not adequately hydrated and nourished [11,12]. Low glycogen content in a fatigued muscle has been found to disrupt myofibril calcium (Ca^2+^), reducing a muscle’s contractility and performance [13]. Furthermore, research has found that decreased muscle glycogen content can alter highly neural activities, such as sprint performance, throughout competitions [14]. While fatigue will likely increase during a preseason training camp, it is unknown how glycogen content and replenishment will be altered as a result of an increased training load and reduced recovery [12]. Utilizing a noninvasive method of measuring muscle glycogen content may be valuable for practiioners to identify fatigue in athletes and to better assess recovery and nutrition.

Therefore, the purpose of the study was to assess s-RPE and high-frequency ultrasound muscle fuel ratings in the right and left RF throughout an intensive nine-day, 16 practice preseason training period in NCAA division I volleyball athletes. We hypothesized that training load would adversely affect muscle fuel ratings throughout the preseason.

## 2. Materials and Methods

### 2.1. Participants

A total of 13 division I female volleyball athletes (18–22 years old) were monitored throughout a full preseason training camp that consisted of 16 total practices across 9 days. Prior to participation, athletes were cleared by the team physician and read and signed an informed consent form prior to participation in the athletic season. The university Institutional Review Board approved the retrospective study and analysis.

### 2.2. Protocol

The study was a repeated measures design so that every athlete was measured daily throughout the study. Before the first practice, right and left RF ultrasounds (Phillips Lumify L12-4 Transducer, Bothell, WA, USA) were recorded, followed by height, weight, standing reach height, and best of three unilateral left, right, and bilateral approach jumps. Approach jumps were measured to assess unilateral and bilateral power. Power was calculated after all jumps were completed; the athlete’s power (in watts) was calculated using the validated Sayers equation (60.7 × height (cm) + 45.3 × body weight (kg) − 2055) [15]. Lastly, participants reported their dominant hand and dominant leg when attempting single-leg approach jumps.

While consuming breakfast was not mandatory, athletes were strongly encouraged to eat carbohydrate-containing foods upon entering the facility. Then, each morning, 30 min before practice, athletes were required to sit in the supine position with both legs straight while the left and right RF ultrasound scans were recorded. On the first day, athletes participated in a conditioning test followed by a morning practice session. After the first practice, athletes were encouraged to eat, drink fluids, and participate in recovery or injury treatment (if needed), and then athletes practiced again later in the afternoon. Except for the conditioning test, the same morning schedule was utilized throughout the preseason. Additional afternoon practice sessions occurred on days 1–3 and then on days 5, 6, and 8. Athletes ate a primary meal immediately after morning and afternoon practice sessions. Throughout the preseason, athletes were provided with food, snacks, and drinks by the university and all athletes were encouraged to consume high-carbohydrate-containing foods and drinks at every meal and snack. Due to the repeated measures design, athletes were allowed to eat or drink ad libitum throughout the preseason phase. The university Institutional Review Board approved the retrospective study and analysis.

### 2.3. Training Load

To assess training load, s-RPE was utilized in the current study. Session RPE is the product of session duration and an athlete’s RPE and is a valid and reliable indicator of internal training load [16,17,18]. Also, s-RPE has been utilized in previous volleyball research to quantify internal training loads throughout 3-, 4-, and 5-set match-play [10]. Throughout the preseason, after each training session, athletes reported their RPE using the validated modified Borg scale that ranged from 0–10, with 10 being maximum-effort activity [17]. When multiple training sessions occurred, s-RPE was summed for the day to provide a single-day training load value. There were a total of nine training days corresponding to nine s-RPE scores for each athlete throughout the study.

### 2.4. High-Frequency Ultrasound

The Lumify transducer was used with water-soluble transmission gel to obtain high-frequency ultrasound images. The images were then analyzed with the validated MuscleSound^®^ software (MuscleSound, Glendale, CO, USA) application [3,19]. To process the short-axis, single-image scans, the software required a total of three scans for the left RF and three for the right RF muscle. The software then processed the left and right scans and calculated a single score for each side, then averaged the two sides for one muscle fuel rating score. The software calculates a muscle fuel rating score by detecting variations in the greyscale ultrasound image. The software isolates the muscle fibers of the RF and calculates the mean pixel intensity (greyscale brightness) of the scans to create a muscle fuel rating score ranging from 0–100. For example, when glycogen is low, the ultrasound image of the muscle tissue is brighter (hyperechoic), and when glycogen is high, the ultrasound image is darker (hypoechoic) [2,19]. Furthermore, the recommended location of the RF measurements were based on the athlete’s height, and then the manufacturer’s guidelines specified a distance above the athlete’s patella to make each measurement to ensure consistent measurements were made for each athlete on a day-to-day basis. When scanning the RF muscles, the software required the top fascia to be bright, horizontal, and continuous (e.g., continuous from the left to right side of the scan screen) and the bottom fascia was required to be bright, continuous, and centered on the scan. Ultrasound recordings were taken each morning, 30 min prior to the first practice session of the day. The same trained ultrasound technician recorded the measurements throughout the study. Utilizing high-frequency ultrasound to assess muscle glycogen content has been validated and used in previous research [19,20].

### 2.5. Statistical Analysis

There was a total of nine days of practice that consisted of 16 total practices for 13 athletes. Descriptive statistics (means and standard deviations) were calculated for physical characteristics and jump performance, training load, and muscle fuel ratings. Data were reported as the mean ± SD with 95% confidence intervals and effect size (η^2^). Multiple correlations were utilized to assess if any relationships existed between s-RPE and changes in muscle fuel rating. Effect sizes for correlations were based on Cohen’s [21] estimation of 0.1 = small, 0.3 = moderate, and 0.5 = large effects. Paired *t*-tests were used to compare left and right jump performance tests at the beginning of the season and muscle fuel ratings for each athlete and as a team throughout the preseason. Then, random effects models were used to assess any major effects of time on s-RPE and muscle fuel ratings. Pairwise comparisons were made for all major effects of time. Lastly, effect sizes for paired *t*-tests were estimated using Cohen’s d (trivial (0–0.19), small (0.20–0.49), medium (0.50–0.79), and large (0.80 and greater)) [21]. All statistics were analyzed using IBM SPSS 24.0 (Version 24.0, IBM Inc., Armonk, NY, USA). The criterion for statistical significance was set a priori at *p* ≤ 0.05.

## 3. Results

### 3.1. Physical Characteristics and Jump Performance

Participants were 19.0 ± 0.9 years old, 178.8 ± 7.1 cm tall, and weighed 65.3 ± 5.7 kg prior to the season. Jump performance results and left and right jump comparisons are provided in Table 1.

### 3.2. Correlations

There was a significant, negative correlation between s-RPE and average muscle fuel ratings (r = −0.277, *p* = 0.004), and another significant, negative correlation between s-RPE and left RF muscle fuel ratings (r = −0.341, *p* < 0.001). There was no relationship between s-RPE and right RF muscle fuel ratings throughout the preseason (r = −0.166, *p* = 0.092). An increase in training load assessed via s-RPE was related to a decrease in average and left RF muscle fuel ratings the following morning.

### 3.3. Nine-Day Team Analysis

As a team, a paired *t*-test revealed a large significant difference between left (51.7 ± 17.9) and right (32.8 ± 17.4) RF muscle fuel ratings (Cohen’s d = 1.08, *p* < 0.001). Random effects analyses revealed a significant major effect of time on s-RPE (F = 39.55, *p* < 0.001, η^2^ = 0.862) and left RF muscle fuel rating (F = 3.64, *p* = 0.001, η^2^ = 0.463), but not average RF muscle fuel rating (F = 1.92, *p* < 0.065, η^2^ = 0.353) and right RF muscle fuel rating (F = 0.56, *p* < 0.811, η^2^ = 0.20, Table 2). Pairwise comparisons for s-RPE showed that training load significantly decreased from the beginning to the end of the training camp. Conversely, left RF muscle fuel ratings significantly increased from the first to the second day and remained greater than the first day all throughout the preseason.

### 3.4. Individual Analysis

**Figure 1:**
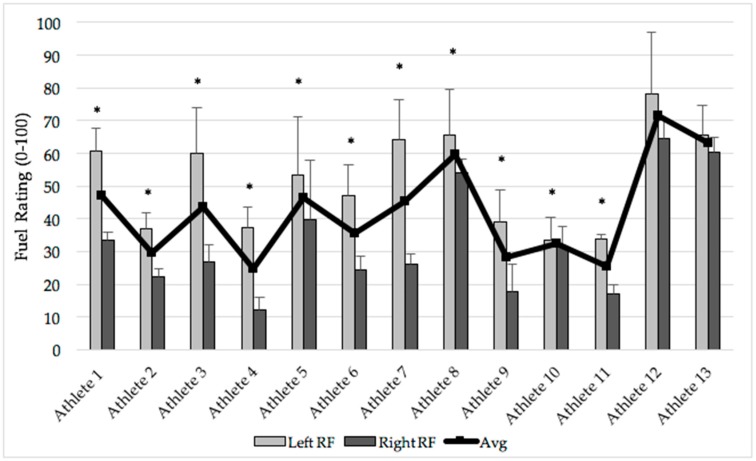
Individual volleyball athlete average, left, and right RF muscle fuel ratings. Data are means ± SD. * Indicates the left RF was significantly greater than the right; *p* ≤ 0.031 for all. Although not significant, Athlete 12 and 13 significance levels were close to reaching significance: *p* = 0.056 and *p* = 0.058, respectively. SD = standard deviation; RF = rectus femoris.

Multiple paired *t*-tests revealed left RF muscle fuel ratings were significantly greater than right RF muscle fuel ratings for 11 of the athletes (*p* ≤ 0.031 for all, Figure 1). All athletes had significantly greater muscle fuel ratings in the left RF when compared to the right RF throughout the preseason.

## 4. Discussion

Contrary to our primary hypothesis, average muscle fuel ratings assessed via high-frequency ultrasound increased 32.2% from the first day to the second day, despite the first and second days being the most intense (i.e., greatest s-RPE) days of the whole preseason. Average muscle fuel ratings then remained elevated after the first day throughout the preseason. A plausible explanation, despite the initial load increase, could be that athletes were provided with ample carbohydrate- and protein-containing foods and drinks while on campus and may not have been adequately nourished prior to the first day of camp. Second, there were unexpected bilateral asymmetries in muscle fuel ratings between the left (dominant) and right (nondominant) RF muscles. As a team, the left RF muscle fuel rating was 57.6% greater than the right, and 11 of the 13 athletes had significantly greater daily left RF muscle fuel ratings. Interestingly, the other two athletes were tremendously close to exhibiting significantly greater left RF muscle fuel ratings throughout the preseason (Table 2). Furthermore, the differences between the left and right RF ultrasound scans suggest that glycogen replenishment or utilization is different between dominant and nondominant legs in volleyball athletes, especially when engaging in high preseason training loads.

Trained, relative to untrained, muscles have been shown to replenish glycogen at a more rapid rate, even when exercise intensity is high and glycogen levels are low [1,22]. Piehl et al. [1] found histochemical estimations of pre-exercise glycogen content to be 32% higher in trained legs relative to untrained legs [1]. Similarly, Kristiansen et al. [22] reported that glucose uptake is 22–33% greater in trained muscles during high-intensity exercise and was likely due to a 66% increase in glucose transport proteins (Glut-4) in the muscle. In the current study, the left-to-right RF comparisons and the correlations between s-RPE and muscle fuel ratings imply a similar phenomenon, supporting improved glycogen replenishment on one side of the body. For example, the negative correlation between s-RPE and the left RF muscle fuel rating was significant and stronger (−0.341) than the nonsignificant right RF ratings (−0.166).

Interestingly, every athlete (13 out of 13) reported to be a right-handed hitter and left-leg dominant. Athletes indicated their left leg to be their primary leg utilized for single-leg attack and approach jumps at the net. While daily single-leg approach jumps were not monitored in the current study, it is likely repetitive dominant, single-leg approach jumps throughout practice and training that result in a dominant-leg muscle that is better conditioned than the contralateral muscle. To support this notion, the athletes’ left legs were also more powerful and could produce a higher approach jump when assessed prior to the first preseason practice. The asymmetries in jump performance may be indicative of asymmetries in muscle fuel ratings between the left and right RF. Again, this concept is consistent with research suggesting that trained muscles can replenish glycogen more efficiently [1,22].

The current results provide foundational data for the use of high-frequency ultrasound to determine bilateral asymmetries in glycogen content in volleyball athletes. Furthermore, the combination of ultrasound and injury data may be a useful tool to assess if imbalances are related to injuries. Comparable data has been reported, suggesting that athletes with large asymmetries in muscle strength and bilateral deficits are more prone to injury [23,24,25]. In addition to asymmetries, low muscle glycogen content is related to a decrease in intramuscular Ca^2+^, which negatively influences excitation–contraction coupling, further affecting the contractile properties of the muscle and its fatigability [13]. Although less is known regarding the effectiveness of utilizing glycogen content asymmetries to predict injury and performance, glycogen content assessments may provide valuable information regarding the internal health of a muscle differently than strength and power tests. Unlike strength and power tests, ultrasound assessments do not require physical effort or strain, are less cumbersome to the athlete, and are not influenced by an athlete’s effort.

The current study provides novel insight regarding bilateral asymmetries in glycogen replenishment between the left (dominant) and right (nondominant) RF muscles in volleyball athletes; however, it is not without limitations. First, glycogen replenishment is largely influenced by carbohydrate ingestion, and athletes were not required to eat and drink a predetermined number of calories, carbohydrates, proteins, and fats each day, nor were they required to eat and drink prior to morning ultrasound scans. Even though athletes served as their own control, it is possible that athletes replenish glycogen content differently if nutrition (i.e., strategic ingestion of carbohydrates and proteins) and hydration (i.e., strategic ingestion of water and sports drinks) protocols are adequate and controlled throughout the day. Additionally, ultrasound assessments of glycogen content scores may be influenced by muscle size, postural alignment [26], and even ultrasound transducer pressure. While the current standard for assessing glycogen content within a muscle is to obtain muscle biopsies, this technique was not feasible during the preseason. Therefore, glycogen content (mmol·kg^−1^) was not compared to ultrasound muscle fuel rating scores out of 100. With regard to training load, jump loads were not monitored throughout practices, especially single-leg approach jumps. It is possible that the quantity of daily jump loads may be a critical factor in the differing replenishment of glycogen in the RF muscles. Lastly, self-reported fatigue and acute jump performance was not assessed in relation to the bilateral asymmetries in RF muscle fuel ratings. Future research should aim to assess if asymmetries in glycogen content assessed via high-frequency ultrasound contribute to self-reported fatigue and altered jump performance.

## 5. Conclusions

In conclusion, high-frequency ultrasound scans revealed that athletes increased RF muscle fuel rating scores despite an initial increase in training load in the first two days of preseason practice. Interestingly, athletes exhibited significantly different muscle fuel ratings between their left (dominant) and right (nondominant) RF muscles throughout the preseason. While it is difficult to ascertain the mechanism for the bilateral asymmetries in glycogen content, the information from ultrasounds may allow practitioners to better assess the glycogen content of an athlete’s muscle. In turn, this information can be utilized to improve fueling recommendations aimed to enhance glycogen stores and, perhaps, optimize recovery. Ultrasound appears to be a noninvasive assessment tool that can determine glycogen replenishment or utilization in the RF. Additional research is warranted to assess the effectiveness of high-frequency ultrasound on muscle glycogen content.

## Figures and Tables

**Table 1:** Approach jump results for all athletes.

n = 13	Bilateral AJ (cm)	Left Unilateral AJ (cm)	Right Unilateral AJ (cm)	Bilateral Power (watts)	Left Unilateral Power (watts)	Right Unilateral Power (watts)
Athletes	58.6 ± 6.6	51.6 ± 6.0 *	47.6 ± 6.5	4461 ± 551	4034 ± 556 *	3791 ± 563

Data are means ± SD. * Significantly greater than unilateral jump off the right foot, *p* = 0.001. SD = standard deviation; AJ = approach jump.

**Table 2:** Muscle fuel ratings (score: 0−100) and session ratings of perceived exertion (s-RPE) across nine days of preseason volleyball.

**Day**	**s-RPE (AU)**		**Average Muscle Fuel Rating**	
	**Mean ± SD**	**95% CI**	**Mean ± SD**	**95% CI**
1	3010 ± 515 *	2732–3288	28.6 ± 13.1	20.0–37.3
2	2984 ± 271 *	2706–3262	37.8 ± 13.7	29.1–46.4
3	1985 ± 661 ^#^	1707–2263	40.3 ± 18.1	31.7–49.0
4	985 ± 587	707–1263	43.5 ± 16.4	34.9–52.2
5	2038 ± 505 ^#^	1761–2316	44.9 ± 16.2	36.3–53.6
6	1378 ± 700	1100–1656	45.9 ± 17.0	37.2–54.5
7	913 ± 296	635–1190	46.8 ± 16.1	38.2–55.5
8	880 ± 295	602–1158	48.2 ± 16.3	39.5–56.8
9	979 ± 193	703–1256	44.4 ± 13.8	35.4–53.4
**Day**	**Left RF Muscle Fuel Rating**		**Right RF Muscle Fuel Rating**	
	**Mean ± SD**	**95% CI**	**Mean ± SD**	**95% CI**
1	30.7 ± 10.9 ^¥^	21.7–39.8	26.5 ± 17.6	16.8–36.2
2	47.2 ± 15.1	38.1–56.2	28.4 ± 18.4	18.7–38.1
3	49.5 ± 18.9	40.5–58.6	31.1 ± 19.5	21.4–40.8
4	54.9 ± 17.6	45.9–64.0	32.2 ± 16.9	22.4–41.9
5	56.2 ± 17.4	47.2–65.3	33.7 ± 17.3	24.0–43.4
6	55.9 ± 17.3	46.8–64.9	35.9 ± 18.3	26.2–45.6
7	57.2 ± 17.4	48.1–66.2	36.5 ± 17.3	26.8–46.2
8	59.2 ± 16.9	50.2–68.3	37.2 ± 17.8	27.4–46.9
9	54.7 ± 15.3	45.3–64.2	34.1 ± 15.4	24.0–44.2

Data are means ± SD with 95% CI. * Indicates s-RPE is significantly greater than days 3–9, *p* < 0.001. ^#^ Indicates s-RPE is significantly greater than days 4 and 6–9, *p* ≤ 0.05. ^¥^ Indicates left muscle fuel rating is significantly less than days 2–9, *p* ≤ 0.012. SD = standard deviation; CI = confidence interval; RF = rectus femoris; s-RPE = session ratings of perceived exertion; AU = arbitrary units.
